# Clinical application of large channel endoscopic decompression in posterior cervical spine disorders

**DOI:** 10.1186/s12891-019-2920-6

**Published:** 2019-11-18

**Authors:** Chengli Li, Xiaojie Tang, Song Chen, Yongchun Meng, Wei Zhang

**Affiliations:** 1 Department of Spine surgery, The Third Hospital of Hebei Medical University, Shijiazhuang, 050051 China; 2 Department of orthopaedics, Yantai Affiliated Hospital of Binzhou Medical College, Yantai, 264100 Shandong Province China

**Keywords:** Cervical vertebrae, Cervical spondylotic myelopathy, Posterior cervical spine, Large-channel endoscopy

## Abstract

**Background:**

We investigated the clinical value of posterior percutaneous endoscopic decompression (PED) for single-segment cervical spondylotic myelopathy (CSM) and cervical spondylotic radiculopathy (CSR). Methods: Clinical data from February 2016 to March 2018 were collected for 32 patients with single-segment CSM or CSR who underwent posterior cervical percutaneous large channel endoscopic decompression and completed a regular follow-up exam at 12 months after surgery. Patient data included: age (range 30–81 years and mean of 49.5 years) and surgical information (operation time, bleeding volume, hospital stay, complications, etc.). The Japan Orthopedic Association (JOA) score and pain visual analog scale (VAS) were used to evaluate the surgical outcome for each patient. Cervical spine radiographs were used to evaluate cervical curvature (Cervical spondylotic angle (CSA), C2–7 Cobb angle) and CT and MRI were used to assess the extent of laminectomy and nerve root decompression. The JOA score, VAS score, cervical curvature were analyzed statistically, and the clinical outcome was evaluated using modified Macnab criteria at the last patient follow-up exam.

**Results:**

The JOA and VAS scores were compared before and after surgery (1 day Pre-op; 3 days, 3 months and 12 months Post-op). The differences were statistically significant (*P* < 0.05). There were significant differences in cervical curvature (C2–7 Cobb angle) between the time points (1 day Pre-op; 3 days, 3 months and 12 months Post-op), but the differences were no statistically significant in CSA angle (*P* < 0.05) The operation time range was 45–110 min (mean 68.6 ± 23.8 min); the intraoperative blood loss range was 20–85 ml (mean28 ± 14.8 ml), and the hospital stay was 3–8 days (mean4.5 days). At the last follow-up, the clinical efficacy was evaluated using modified Macnab criteria. The results were excellent in 18 cases, good in 11 cases, and fair in 3 cases. The combined excellent and good rate was 93.75%. Postoperative CT and MRI showed that the compression of the spinal cord or nerve roots was completely relieved.

**Conclusion:**

Endoscopic decompression of posterior cervical vertebral disorders is a safe, effective, and minimally invasive surgical procedure with rapid recovery times. This procedure warrants additional research and clinical application.

## Background

Cervical spondylotic myelopathy (CSM) or cervical spondylotic radiculopathy (CSR) are the most common types of cervical spondylosis, often requiring surgical intervention. Surgical treatment is especially important after a definitive diagnosis of CSM [1]. Traditional open surgery of the posterior cervical spine is associated with a range of serious complications, including paralysis of paravertebral muscle tissue and significant bleeding and as well as others. The post-intrinsic axial symptoms [2–4] of posterior cervical surgery greatly affect the postoperative results and the patients’ satisfaction with the surgical outcome. Traditional cervical anterior decompression and fusion surgery is currently considered to be standard surgical procedure for degenerative cervical vertebral disease [5], due to its relative safety, a reasonable degree of clinical efficacy and minimally invasive approach, However for “clamped” single-segment cervical spondylotic myelopathy, spinal cord compression emanates from the anterior and posterior sides, making it difficult to relieve the compression using the anterior approach alone, typically elderly patients do not tolerate anesthesia and open trauma well, suggesting that alternate surgical methods to traditional surgical decompression should be evaluated. For these reasons, we selected 32 patients diagnosed with cervical spondylosis and who underwent posterior cervical percutaneous large channel endoscopic decompression for treatment to evaluate the surgical results. In the study we summarized the early clinical efficacy and the possible complications associated with using cervical PED to treat cervical spondylosis; clarified the clinical value of and precautions needed for PED; and explored the surgical indications and limitations of PED.

## Methods

### Patients

From February 2016 to March 2018, we evaluated 32 patients diagnosed with single-segment CSM or CSR in the Spinal Surgery of our Hospital and treated with posterior cervical percutaneous large channel endoscopic decompression (PED). The patient ages ranged from 30 to 81 years (mean age was 49.5 years). The duration of symptoms ranged from 1 month to 2 years, with a mean of 5 months. Seven cases of cervical spondylotic myelopathy were included that primarily resulted in numbness and weakness of the limbs, and three of these patients also experienced neck and shoulder pain. Twenty-five cases of cervical spondylotic radiculopathy were included that mainly resulted in the patients experiencing unilateral limb neck and shoulder pain or upper extremity radiating pain. All 32 patients underwent cervical anterioposterior and lateral radiographs, dynamic position radiography, as well as CT and MRI.

Inclusion criteria: I. Clinical diagnosis of single-segment cervical spondylotic myelopathy (CSM) or cervical spondylosis radiculopathy (CSR). II. All patients underwent posterior cervical percutaneous large channel endoscopic decompression. Of these 32 cases, thirteen patients underwent combined anterior cervical decompression and fusion, and PED was regarded as a supplemental procedure or as preparation for open anterior surgery. Exclusion criteria: I. Excluding cervical instability, and the lateral cervical radiography and the dynamic position radiography revealed obvious kyphosis, II. The presence of a cervical vertebral tumor, or possible infection, III. Cervical spine fracture.

### Surgical methods

**Fig. 1:**
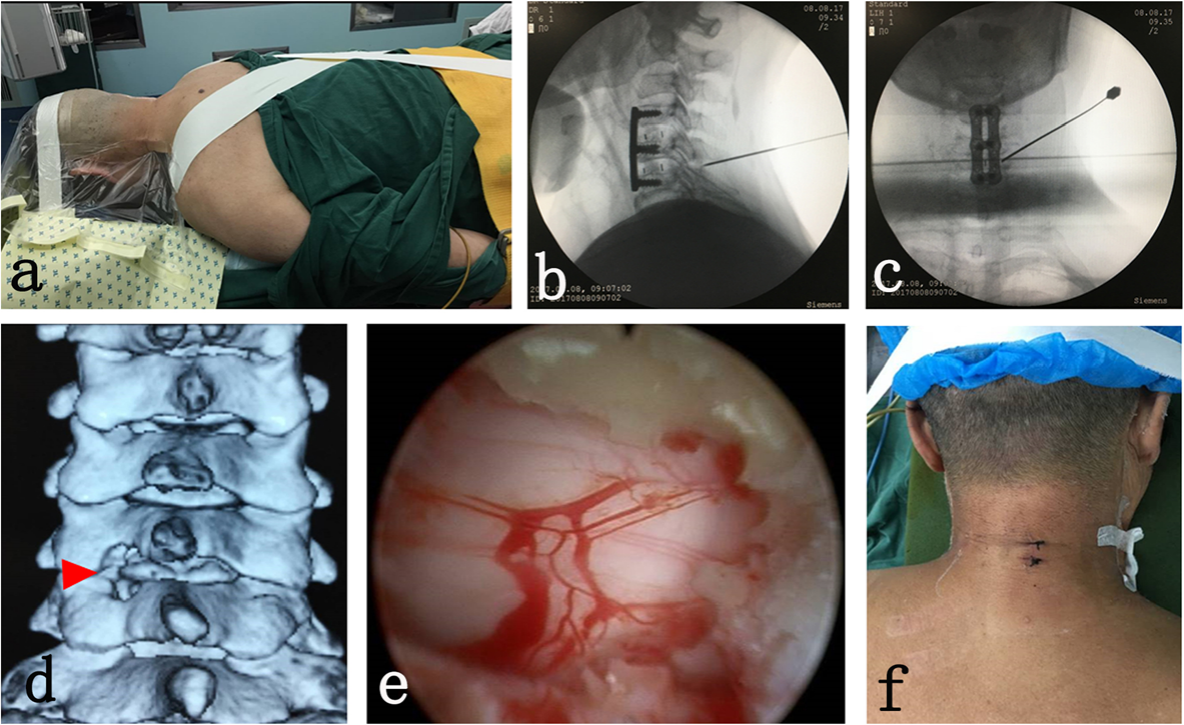
**a** The high head and low foot placement; **b** The 8G long needle was positioned the corresponding surgical gap laterally; **c** On the positive, the needle faced the medial edge of the superior and inferior articular processes; **d** The “V” point of the anatomical landmark; **e** The posterior compression lesion was completely decompressed; **f** The incision was closed using 1 or 2 sutures

After general anesthesia with tracheal intubation, each patient was placed in the prone position with a high head and low foot placement (Fig. 1a). The cervical vertebrae was fixed with a specific head fixator and wide tape. The shoulders were pulled toward the lower back with wide tape for intraoperative positioning.

The posterior decompression surgery segment was positioned using fluoroscopy with a C-wall X-ray machine. Along the posterior medial side of the cervical vertebra, about 1.5 cm apart, an 8G long needle was positioned at the corresponding surgical gap laterally (Fig. 1b), and for the positive placement, the needle faced the medial edge of the superior and inferior articular processes (Fig. 1c), The longitudinal incision along the tip of the needle was approximately 10 mm, The working channel was placed into the incision, and then the endoscope was placed, and the correct channel position was determined. Soft tissue around the “V” point was cleaned to clearly expose the “V” point (Fig. 1d) of the anatomical landmark. If hyperplasia was obvious, the hyperplastic bone was removed first, using gun bone-biting forceps and then the upper lamina was partially removed medially along the “V” point to expose the origin of the ligamentum flavum. Next, the lower lamina was partially removed using gun bone-biting forceps to expose the insertion of the ligamentum flavum on the lower lamina, the ligamentum flavum was removed upward along the insertion point. Attention was focused on the attachment between the ligamentum flavum and the dural sac during debridement to avoid damage to the dura mater. Parts of the superior and inferior articular processes were removed on the lateral side, and the area of the removed articular process to the outside was less than 50% of the articular process. During the procedure, nerve probes were used to distinguish the nerve root from the dura mater and the intervertebral disc to avoid damage to the nerve root. Under the endoscope the posterior compression lesion was completely decompressed (Fig. 1e). After decompression of the spinal cord or nerve root, the endoscope and cannula were removed and 1 or 2 sutures were used to close the incision (Fig. 1f). Unilateral or bilateral decompression was accomplished according to the needs of the specific patient, and the operation time, bleeding volume and any complications were recorded.

### Postoperative treatment

Approximately 12 to 24 h after surgery, the patients were able to resume moderate activity with adequate protection from a neck support. Additional treatments such as intraoperative nerve root and spinal cord traction, dehydration drugs, and hormones were used postoperatively up to 3 days for some patients. Antibiotics were not routinely used before or after surgery. According to the degree of postoperative pain, the appropriate amounts of analgesic drugs were applied, and the length of postoperative recovery time before returning to normal work depended on the specific circumstances for each patient. The sutures were removed 7 to 10 days after surgery.

### Data collection

#### General patient data

Perioperative data were collected for each patient including, duration of surgery, amount of bleeding, any complications, improvement of postoperative symptoms, follow-up observations of postoperative symptoms, and follow-up imaging using radiography, MRI, or CT.

#### Clinical observation indicators

JOA (Japan Orthopedic Association) and VAS (visual analogue scale) scores were performed at various time points before and after surgery (1 day Pre-op; 3 days, 3 months, and 12 months Post-op) to evaluate the improvement of cervical nerve function and the extent of neck or upper limb pain. JOA Score was a method used to evaluate the spinal cord function of patients with cervical spondylosis, which was developed by Japan Orthopedic Association (JOA) on March 18, 1994 after several revisions, the scores range 0–17, included the upper and lower limb motor function, sensory impairment and bladder function. The higher score indicated the better function of the cervical spine, the greater difference between pre-op and post-op indicated the better effect of the operation. The VAS score range 0–10, 0 show painless, 10 show intolerance of severe pain, and the middle part show varying degrees of the pain. The final follow-up exam included patient evaluation using the modified Macnab criteria for clinical efficacy.

#### Imaging observation indicators

**Fig. 2:**
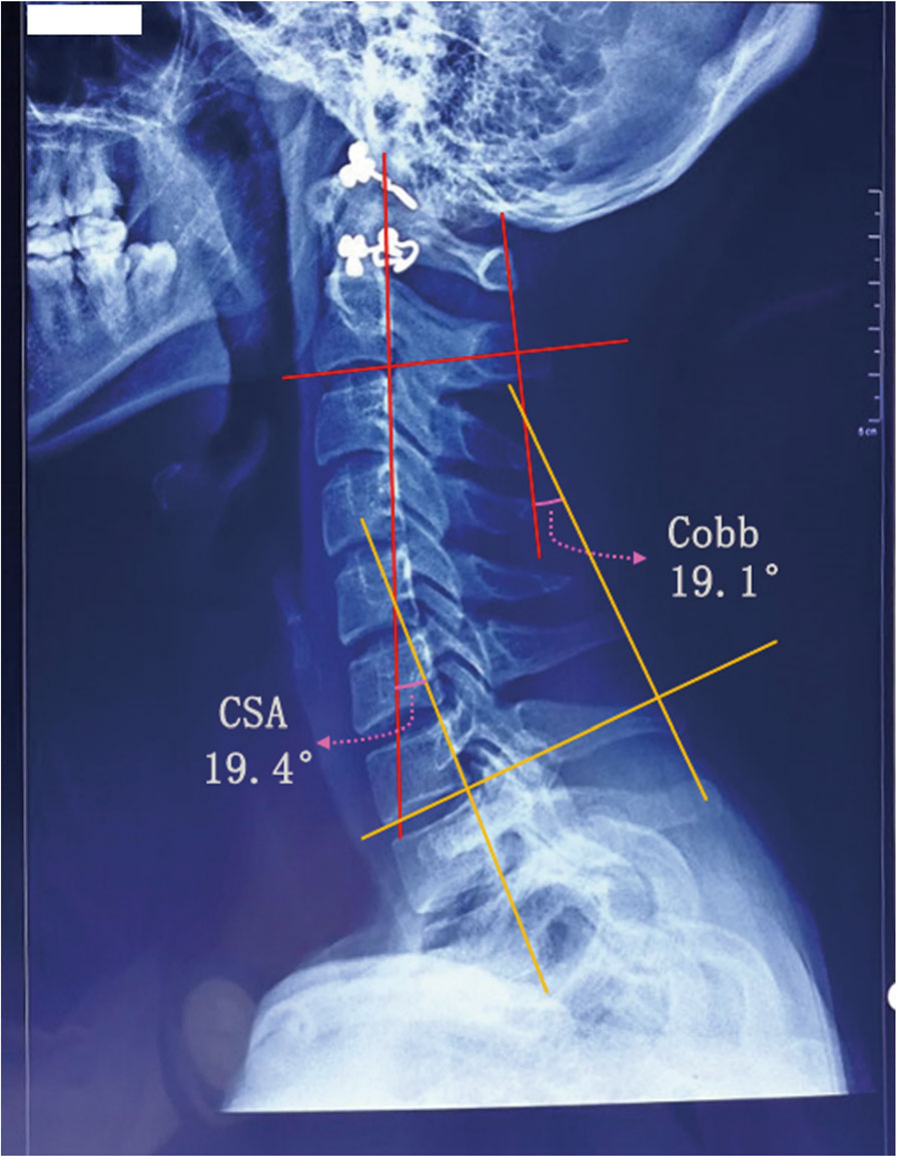
The measurement of CSA angle and Cobb angle. Jackson physiological curve; a line was drawn parallel to the trailing edge of C2 and C7, respectively, and the angle between them (CSA) represented the cervical curvature. The Cobb angle four-line measurement: the first line was parallel to the end plate of C2, the second line was parallel to the end plate of C7; two vertical lines were drawn for the above two lines, and the angle between the two perpendicular lines was the Cobb angle

Cervical radiographs (Fig. 2) were performed at various time points before and after surgery (1 day Pre-op; 3 days, 3 months, and 12 months Post-op) to evaluate changes in cervical curvature. To determine the Jackson physiological curve [6]; a line was drawn parallel to the trailing edge of C2 and C7, respectively, and the angle between them (CSA) represented the cervical curvature (Fig. 2). The Cobb angle four-line measurement [6] was carried out in which the first line was parallel to the end plate of C2, the second line was parallel to the end plate of C7, two vertical lines were drawn for the above two lines, and the acute angle between the two perpendicular lines was the Cobb angle (see Fig. 2). The positive value of C2–7 Cobb angle showed that the cervical curvature was “lordosis” and the negative value showed that the cervical curvature was “kyphosis”. Normal range of C2–7 Cobb angle; Age ≤ 55 old, Male 22.74 ± 4.23^o^, Female 21.39 ± 5.28^o^, Age ≥ 56 old, Male 20.16 ± 3.51^o^, Female 20.16 ± 4.13^o^. The C2–7 Cobb angle was more close to 0, showing that the cervical curvature tends to be more straight.

**Fig. 3:**
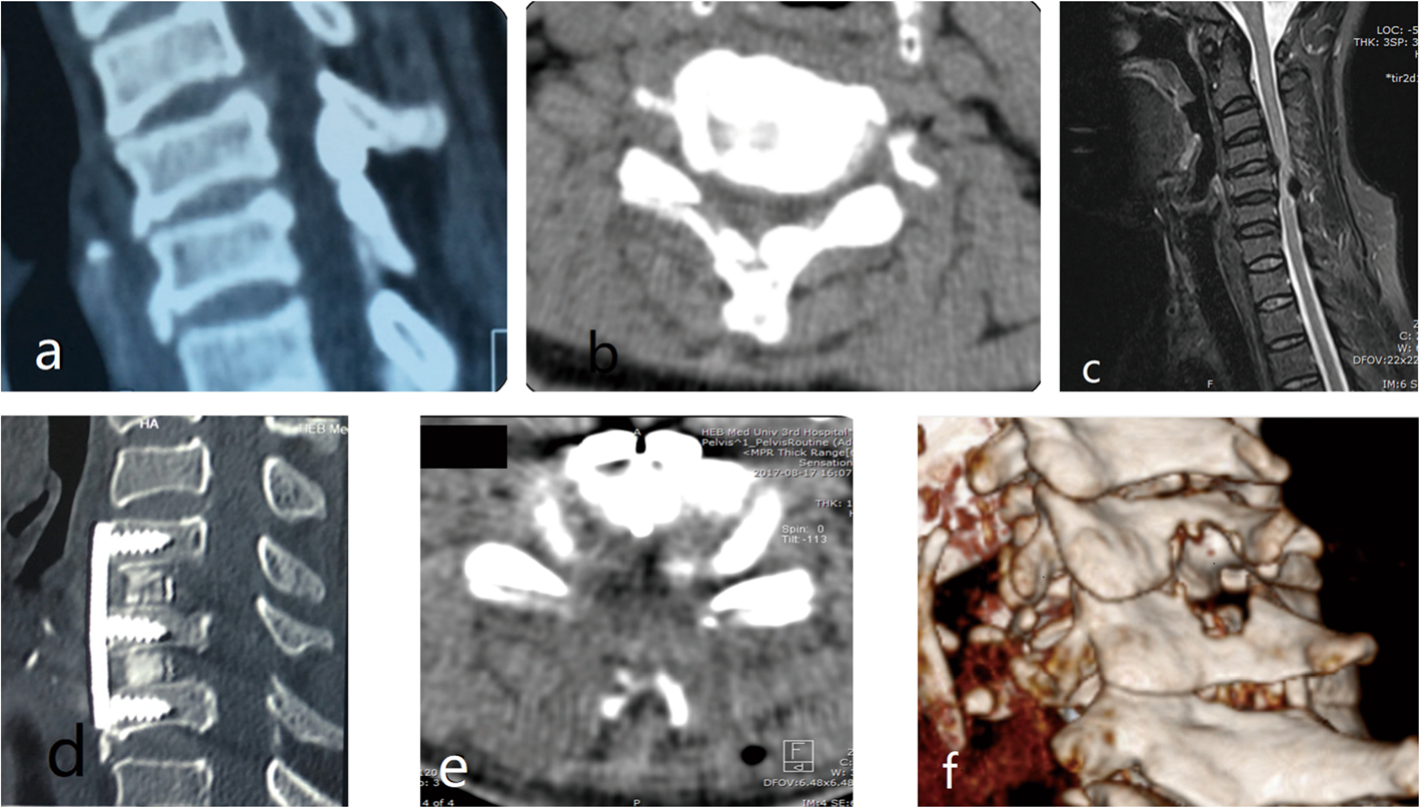
Preoperative and Postoperative Imaging Comparison. Preoperative cervical CT and MRI (**a**, **b**, **c**) showed C4–5 and C5–6 disc herniation with calcification of C5 posterior ligamentum flavum, spinal stenosis and spinal cord compression. In the first stage, anterior cervical decompression and fusion were performed, and the clinical symptoms were not improved thoroughly. In the second stage, bilateral lamina decompression was performed under endoscopy to remove the compression from C5–6 posterior (**d**, **e**, **f**).

CT and MRI were performed 3 to 7 days after surgery to evaluate spinal cord signals and changes associated with decompression (Fig. 3). The cervical vertebral MRI was taken 6 to 12 months after surgery to evaluate any changes in the anatomical relationship of the posterior muscles.

### Statistical processing

Data were entered into SPSS 19.0 software for analysis. The measurement data were expressed as means ± standard deviation. The paired variance t-test was used, and comparisons between preoperative and postoperative scores were made. When *P* < 0.05 was achieved, the difference between the two groups was considered to be statistically significant. To express the statistical differences between two groups more intuitively, a histogram was designed to show the comparisons.

## Results

### Surgical results

Thirty-two patients underwent endoscopic surgery for posterior cervical vertebral disorders. The time needed for surgery ranged from 45 to 110 min, with a mean of 68.6 ± 23.8 min. The intraoperative blood loss ranged from 20 to 85 ml, with a mean of 28 ± 14.8 ml. The hospital stay ranged from 3 to 8 days, with a mean of 4.5 days, and the postoperative follow-up time was 6 to 10 months.

### Cervical spinal cord function JOA score

**Fig. 4:**
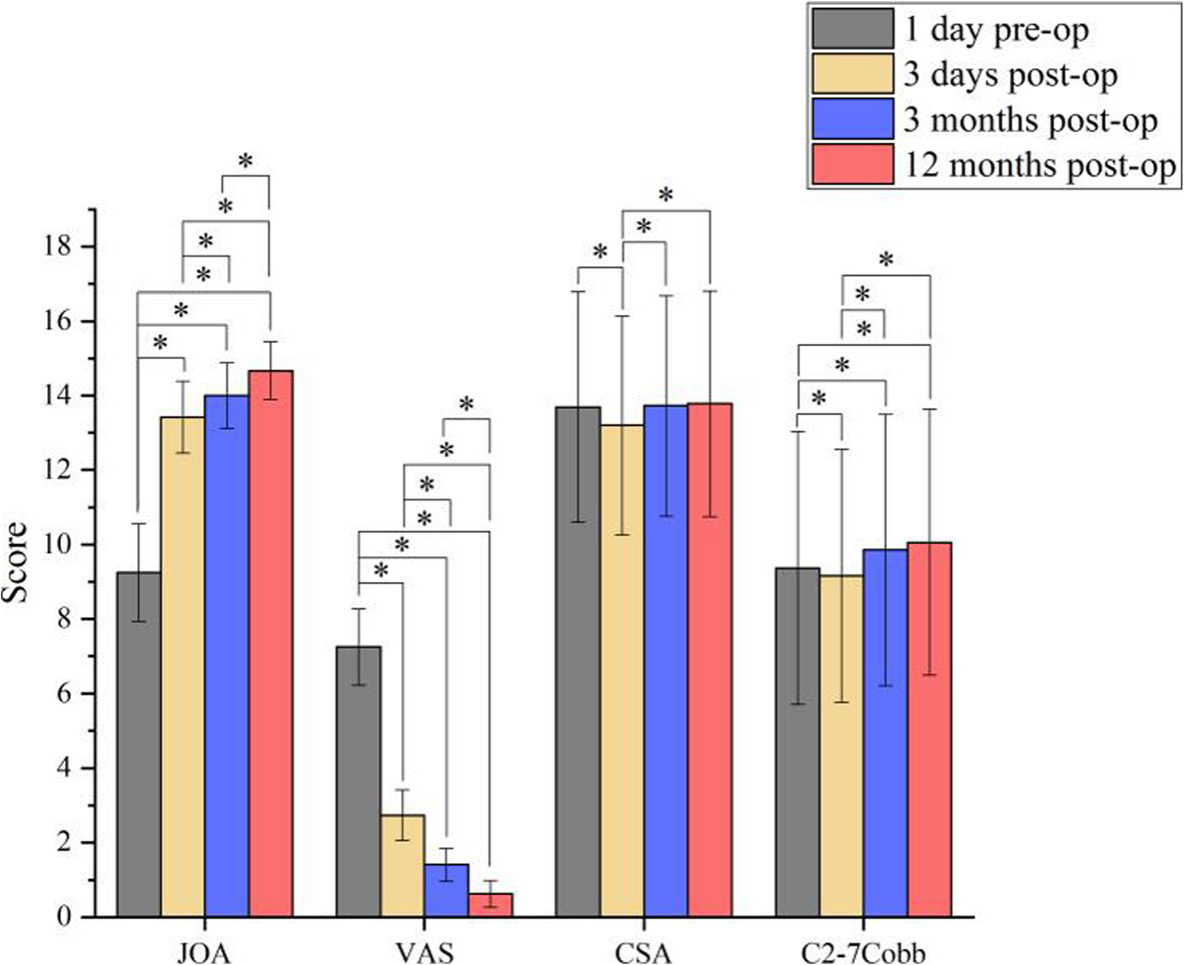
A Histogram Comparison among Scores. (*show that there was statistical significance between the scores of the two groups. When *P* < 0.05)

The JOA score on the 3rd postoperative day was significantly higher than the score observed on the preoperative day. The JOA score increased gradually at 3 months and 12 months postoperative. The differences were significant compared to the score on the preoperative day (*P* < 0.05, Table 1, Fig. 4). These results demonstrate that the symptoms of cervical spondylotic myelopathy or radiculopathy were alleviated and function gradually improved.

**Table 1:** Japan Orthopedic Association (JOA) score results

Time	1 day pre-op	3 days post-op^a^	3 months post-op^b^	12 months post-op^c^
JOA score	9.25 ± 1.31	13.42 ± 0.97	14.0 ± 0.88	14.67 ± 0.78

JOA scores are presented as mean number ± standard deviation ($\overline{x}$ ±s, *n* = 32). “^a b c^” respectively represents the comparison between 3 days, 3 months and 12 months post-op and pre-op. Analysis using paired t-tests showed that P_a_、P_b_、P_c_<0.001, indicating significant differences were observed

### Reduction of neck or upper limb pain

The VAS score was significantly lower on postoperative day 3 compared to the preoperative score, and the VAS score decreased gradually at 3 and 12 months postoperative. The differences were statistically significant compared to the preoperative VAS score (P < 0.05, Table 2, Fig. 4). Thus, the symptoms of the neck or upper limbs pain were significantly relieved following the surgical procedure, and the pain gradually improved during the follow-up period.

**Table 2:** Visual analogue scale (VAS) score results

Time	1 day pre-op	3 days post-op^a^	3 months post-op^b^	12 months post-op^c^
VAS score	7.25 ± 1.02	2.73 ± 0.68	1.40 ± 0.44	0.63 ± 0.36

VAS scores are presented as mean number ± standard deviation (x ®±s, *n* = 32). “^a b c^” respectively represents the comparison between 3 days, 3 months and 12 months post-op and pre-op. Analysis using paired t-tests showed that P_a_、P_a_、P_c_ = 0.000<0.001, indicating significant differences were observed

### Evaluation of surgical efficacy

At the last follow-up, the clinical efficacy was evaluated using modified Macnab criteria. The results were excellent in 18 cases, good in 11 cases, and fair in 3 cases. The combined excellent and good rate was 93.75%.

### Surgical imaging comparison

**Fig. 5:**
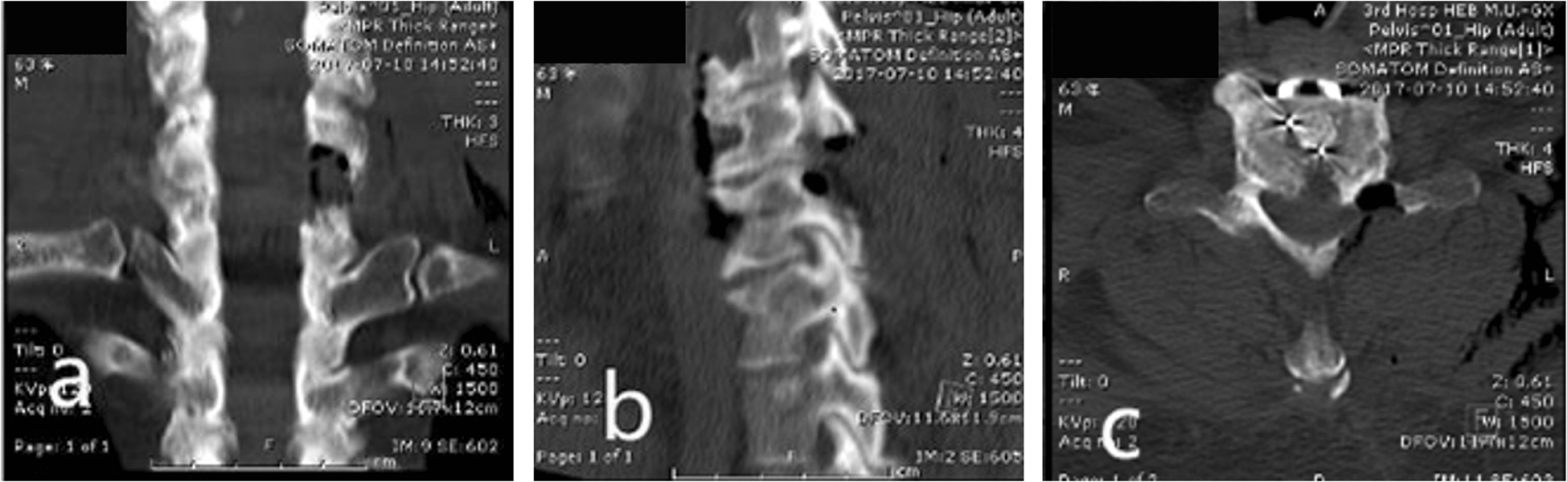
The CT imaging changes after cervical6–7 Key-hole surgery. **a** coronal, **b** sagittal, **c** transverse

There were no significant differences in the CSA or C2–7 Cobb angles obtained from radiograph taken at each evaluation time point (3 days; 3 months and 12 months Post-op) compared to the 1 day preoperative assessment (*P* > 0.05). Table 3 show that significant differences were observed on 3 days postoperative, but there was no statistical significance on 3 months and 12 months postoperative. Table 4 show that significant differences were observed. These results indicated that some patients with cervical curvature may show some improvement, and the large channel endoscope did not affect the stability of the cervical vertebra. The postoperative CT and MRI revealed that the compression of spinal cord or nerve root was completely relieved (Figs. 3 and 5).

**Table 3:** Cervical spondylotic angle (CSA) score results

Time	1 day pre-op	3 days post-op^a^	3 months post-op^b^	12 months post-op^c^
CSA score	13.69 ± 3.09	13.20 ± 2.94	13.73 ± 2.96	13.78 ± 3.02

CSA scores are presented as mean number ± standard deviation (x ®±s, *n* = 32). “^a b c^” respectively represents the comparison between 3 days, 3 months and 12 months post-op and pre-op. Analysis using paired t-tests showed that P_a_ = 0.001、P_b_ = 0.724、P_c_ = 0.561, indicating significant differences were observed on 3 days postoperative, but there was no statistical significance on 3 months and 12 months postoperative

**Table 4:** Cervical curvature (C2–7 Cobb) score results

Time	1 day pre-op	3 days post-op^a^	3 months post-op^b^	12 months post-op^c^
C2–7 Cobb score	9.37 ± 3.66	9.16 ± 3.39	9.86 ± 3.64	10.06 ± 3.57

C2–7 Cobb scores are presented as mean number ± standard deviation (x ®±s, n = 32). “^a b c^” respectively represents the comparison between 3 days, 3 months and 12 months post-op and pre-op. Analysis using paired t-tests showed that P_a_ = 0.046、P_b_ = 0.000、P_c_ = 0.001, indicating significant differences were observed

### Complications

Two patients experienced short-term numbness on one side of their body, but muscle strength was not decreased. After symptomatic drug treatment, the patients gradually improved. Cervical vertebral MRI showed no obvious local hematoma in these patients, and the postoperative hemorrhage also was excluded. Therefore, we conclude that the above symptoms were likely caused by the intraoperative traction of the nerve root. None of the patients experienced any permanent spinal cord or nerve roots damage, important vascular injury, or wound infection.

## Discussion

### Clinical advantages of posterior cervical percutaneous large channel endoscopy

The large channel endoscopic technique is based on the posterior translaminar total endoscopic technique proposed by Ruetten et al [7] The working channel diameter is 10 mm, which is the largest channel diameter in all spine endoscopic techniques described in the literature. The wider endoscope field of view and operating space can be applied to use larger size grinding bits, gun bone-biting forceps, and nucleus pulposus forceps, thus allowing more convenient surgical access.

The large channel endoscope accurately dissects and locates the “V” point under the microscope, avoids the need to peel back the posterior semi-spine muscles, reduces damage to the upper and lower small joint capsules and spinous processes, and retains cervical muscle strength postoperatively [8]. Large channel endoscopy significantly reduces the incidence of adverse postoperative axial symptoms of the neck [9, 10], specifically reducing the complications of postoperative neck stiffness and motor function loss [2–4]. Due to its minimally invasive features [11], large channel endoscopy has been widely used in the treatment of lumbar spinal stenosis. For example, Chiu et al. [12] reported the application of spinal endoscopy to treat lumbar spinal stenosis and reported an excellent plus good rate of 94%.

There is no important neurovascular tissue in the path used with the posterior approach of the large channel endoscope, which avoids damage to important tissues such as the esophagus, larynx, recurrent nerve, vertebral artery, and cervical vessels that are in the path of the anterior cervical endoscope [13]. Large channel endoscopes do not require decompression through the intervertebral disc or vertebral body [14, 15], which avoids endplate inflammation and vertebral height reduction [16] due to surgery, and posterior endoscopic surgery has less adverse effects on cervical curvature and stability [17]. Kim CH [18] reported that when the cervical lordosis angle is less than 10^o^, some patients with cervical curvature may show some improvement. Chao Liu [19] reported good clinical efficacy and safety of posterior cervical endoscopic approach for the treatment of cervical disc herniation. Wang Peng [20] applied a 6.9 mm diameter endoscopic through posterior cervical approach for cervical spondylotic radiculopathy surgeries that also achieved favorable results with an excellent rate of 91.7%. The excellent plus good rate for large channel endoscopy in our hospital for cervical spondylosis was 93.75%.

### Difficulties and limitations of large channel endoscopic techniques

Intraspinal venous plexus hemorrhage is an important consideration in cervical spinal surgery. The posterior venous plexus [21] is located in the anterior epidural fat of the vertebral arch and ligamentum flavum. The plexus is irregular and there are abundant lateral anastomoses. The venous plexus in the spinal canal may be connected with the extraspinal vein behind the vertebral arch through the intralaminar vein and the intravenous vein of the ligamentum flavum. Based on the above anatomy, the large channel endoscope has a high likelihood of causing intraspinal venous plexus hemorrhage during the posterior decompression process. Methods by which we can stop bleeding quickly and effectively need to be considered in our clinical practice. In Ye Xiaojian’s report [22], he mentioned that a radiofrequency cutter head could be used to stop bleeding. In one of the reported cases, intraoperative bleeding led the authors to change to anterior open surgery. Due to continuous intraoperative high water pressure, although hemostasis exists, there is still a potential risk of injury to the spinal cord. However, there are no specific reports concerning how to quantify the perfusion water pressure that is used and reduce potential spinal cord injury.

To correctly determine the range of decompression during operation, first, the “V” point must be accurately located, which is the junction of the lateral upper and lower lamina and the medial aspect of the face. Laminectomy and decompression along the medial side of the “V” was feasible when the spinal stenosis was biased to the center. Then part of the spinous process could be removed for decompression or bilateral laminectomy could be performed for decompression. When the nerve root is compressed to the lateral side, some of the articular processes need to be removed to achieve decompression. A commonly held view [23] among surgeons is that if the extent of the excision is less than 50%, the lateral stability of the vertebral body can be preserved, and the decompression effect can be achieved. The angle between the nerve root and the dural sac is gradually enlarged from top to bottom, and the length of the nerve root is also gradually enlarged. The relationship between the intervertebral disc and the nerve root in each segment also is different, and surgeons need to be knowledgeable concerning the anatomical relationships of the cervical vertebrae, to be able to achieve accurate decompression, and avoid damage to the nerve roots and spinal cord.

Thus, the limitations of the large channel endoscope are manifested in the possible complications that accompany its use. The main complications [13] include nerve root injury, postoperative paresthesia, dural sac injury, or cerebrospinal fluid leakage resulting from tearing of the dural sac. Moreover, there is a risk of full spinal cord damage due to administration of local anesthesia.

### Surgical indications

Endoscopic decompression of the posterior cervical vertebrae is relatively limited in its application. The technique is most widely used to alleviate the lateral, soft protrusion compression of a nerve root and narrowing of the cervical intervertebral foramen [24, 25]. Based on our own clinical experience, the indications for surgical intervention using large channel endoscopy are summarized as follows: I. A cervical intervertebral disc protrudes into the lateral posterior intervertebral space and compresses the nerve root, especially with the high or low cervical vertebrae, which are difficult to expose with the anterior approach. II. Spinal canal stenosis of 1 or 2 segments leading to cervical spondylotic myelopathy. III. Intervertebral foramen stenosis of 1 or 2 segments caused by nerve root-type cervical spondylosis. IV. Combined with a traditional anterior approach as an auxiliary surgical approach. V. No obvious compression was visible with imaging, and but the nerve root block was positive.

The main contraindications for surgery [26, 27] are: I. Multiple segmental cervical spondylotic myelopathy, II. Multiple ossifications of the posterior longitudinal ligament. III. Central type cervical disc herniation combined with a wide base. IV. Obvious cervical instability.

In summary, endoscopic decompression of the posterior cervical vertebrae is a safe, minimally invasive, and effective treatment. Although it may not be the ultimate treatment for cervical spondylosis, it is a step in the treatment of cervical spondylosis. It has become an effective surgery to deal with some of the “local hotspot problems” of the posterior cervical spine, especially when aided by a computerized navigation system or 3D printing technology. This surgical technique should find broader application in the future.

## Conclusion

Endoscopic decompression of posterior cervical vertebrae is a safe, feasible and effective surgical method, with the characteristics of minimally invasive, good curative effect and quick recovery. It is worthy of further researches and promotion in clinical work.

## Data Availability

The datasets analyzed during the current study are available from the corresponding author on reasonable request.

## Abbreviations

C2–7Cobb: Cervical 2–7 Cobb (Cobb angle: named after the American orthopedic surgeon John Robert Cobb)
CSA: Cervical spondylotic angle
CSM: Cervical spondylotic myelopathy
CSR: Cervical spondylotic radiculopathy
JOA: Japan Orthopedic Association
PED: Percutaneous endoscopic decompression
post-op: Postoperative
pre-op: Preoperative
VAS: Visual analogue scale
